# Abdominal Visceral-to-Subcutaneous Fat Volume Ratio Predicts Survival and Response to First-Line Palliative Chemotherapy in Patients with Advanced Gastric Cancer

**DOI:** 10.3390/cancers15225391

**Published:** 2023-11-13

**Authors:** Giacomo Aringhieri, Gianfranco Di Salle, Silvia Catanese, Caterina Vivaldi, Francesca Salani, Saverio Vitali, Miriam Caccese, Enrico Vasile, Virginia Genovesi, Lorenzo Fornaro, Rachele Tintori, Francesco Balducci, Carla Cappelli, Dania Cioni, Gianluca Masi, Emanuele Neri

**Affiliations:** 1 Academic Radiology, Department of Translational Research, University of Pisa, Via Roma 67, 56126 Pisa, Italy; giacomo.aringhieri@unipi.it (G.A.); racheletintori@hotmail.com (R.T.); dania.cioni@unipi.it (D.C.); emanuele.neri@unipi.it (E.N.); 2 Italian Society of Medical and Interventional Radiology, SIRM Foundation, Via della Signora 2, 20122 Milano, Italy; 3 Department of Translational Research on New Technologies in Medicine and Surgery, University of Pisa, 56126 Pisa, Italy; catanesesilvia@gmail.com (S.C.); caterina.vivaldi@unipi.it (C.V.); f.balducci@studenti.unipi.it (F.B.); gianluca.masi@unipi.it (G.M.); 4 Unit of Medical Oncology, Azienda Ospedaliero-Universitaria Pisana, Via Roma 67, 56126 Pisa, Italy; francesca.salani@santannapisa.it (F.S.); m.caccese@ao-pisa.toscana.it (M.C.); e.vasile@ao-pisa.toscana.it (E.V.); virgi.genovesi@gmail.com (V.G.); l.fornaro@ao-pisa.toscana.it (L.F.); c.cappelli@ao-pisa.toscana.it (C.C.); 5 Translational Medicine PhD Course, Institute of Life Sciences, Scuola Superiore Sant’Anna, 56127 Pisa, Italy; 6 Diagnostic and Interventional Radiology, University Hospital of Cisanello, Azienda Ospedaliero-Universitaria Pisana, 56126 Pisa, Italy; s.vitali@ao-pisa.toscana.it

**Keywords:** stomach neoplasms, tomography, X-ray computed, body composition, sarcopenia, disease-free survival

## Abstract

**Simple Summary:**

This study was undertaken to address a crucial issue in the treatment of advanced gastric cancer (aGC). The prognosis for aGC patients is typically poor, with various factors affecting survival, such as disease stage and performance status. However, the impact of body composition, particularly abdominal fat distribution, on aGC patient outcomes remains debated. This research aimed to determine the significance of specific body composition parameters (BCPs), including visceral and subcutaneous fat volumes and the visceral-to-subcutaneous (VF/SF) fat volume ratio, in predicting overall survival (OS) and progression-free survival (PFS) in aGC patients treated with first-line palliative chemotherapy. The findings suggest that the VF/SF volume ratio, as measured by radiological methods, is a robust and independent predictor of survival and chemotherapy response in aGC. This research provides valuable insights into tailoring treatment strategies for aGC patients, potentially impacting clinical decisions and enhancing patient outcomes in the broader medical community.

**Abstract:**

Prognosis in advanced gastric cancer (aGC) is predicted by clinical factors, such as stage, performance status, metastasis location, and the neutrophil-to-lymphocyte ratio. However, the role of body composition and sarcopenia in aGC survival remains debated. This study aimed to evaluate how abdominal visceral and subcutaneous fat volumes, psoas muscle volume, and the visceral-to-subcutaneous (VF/SF) volume ratio impact overall survival (OS) and progression-free survival (PFS) in aGC patients receiving first-line palliative chemotherapy. We retrospectively examined CT scans of 65 aGC patients, quantifying body composition parameters (BCPs) in 2D and 3D. Normalized 3D BCP volumes were determined, and the VF/SF ratio was computed. Survival outcomes were analyzed using the Cox Proportional Hazard model between the upper and lower halves of the distribution. Additionally, response to first-line chemotherapy was compared using the χ^2^ test. Patients with a higher VF/SF ratio (*N* = 33) exhibited significantly poorer OS (*p* = 0.02) and PFS (*p* < 0.005) and had a less favorable response to first-line chemotherapy (*p* = 0.033), with a lower Disease Control Rate (*p* = 0.016). Notably, absolute BCP measures and sarcopenia did not predict survival. In conclusion, radiologically assessed VF/SF volume ratio emerged as a robust and independent predictor of both survival and treatment response in aGC patients.

## 1. Introduction

Gastric cancer is the fifth most common malignancy worldwide and the third leading cause of cancer-related mortality as of 2018 (Thrift et al., 2020). Its prognosis is strongly influenced by the stage at diagnosis. Despite recent advances, the prognosis of advanced (unresectable or metastatic) disease remains poor, and palliative chemotherapy remains the mainstay of treatment [1]. Diagnosis of distant disease is associated with a 5-year survival rate of <5%, with no improvement since 2000 [2].

Advanced gastric cancer (aGC) has different predictors of progression-free (PFS) and overall survival (OS) compared to early gastric cancer. Survival of aGC patients has been shown to depend on stage [3], metastatic sites [4], baseline and pre-operative blood parameters like the neutrophil-to-lymphocyte ratio (NLR) [5], and body composition parameters (BCPs) [6]. Indirect measurements of muscle and fat metrics can be cost-effectively obtained through routine Computed Tomography (CT) examinations. Two-dimensional (2D) CT surrogates of sarcopenia [7] have been recognized as predictors of post-operative complications and outcomes in non-metastatic GC, but their utility in aGC is hampered by the non-specific effects of systemic inflammation and tumor dissemination [8]. Previous work by our group has shown the prognostic impact in aGC of 2D fat measurements, such as the ratio of visceral and subcutaneous fat areas (VFA/SFA) measured at the third lumbar vertebra through CT. Patients with a higher VFA/SFA ratio were shown to have worse PFS and OS compared to the rest of the cohort. This is consistent with the known metabolic disruption occurring in cancer, leading to alterations in body fat distribution [9]. However, 2D parameters have intrinsic limitations: their site of measurement (L3) is conventional but arbitrary and might not account for interpersonal variations in fat distribution. Yet, the use of 3D BCPs for prognosis is less common to date, although it has been investigated in several settings, including pancreatic [10] and endometrial cancer [11].

To our knowledge, no study has so far investigated the prognostic value of radiologically measured 3D BCPs in aGC. The aim of this study is to assess their predictive value with respect to survival and toxicity in a homogeneous cohort of aGC patients treated with standard first-line palliative chemotherapy.

## 2. Materials and Methods

### 2.1. Study Population

Medical records of *N* = 65 patients (20F + 45M) with histologically proven diagnosis of adenocarcinoma of the esophagogastric junction and the stomach from the Azienda Ospedaliera Universitaria Pisana were retrospectively examined. Inclusion and exclusion criteria, treatment administration, and criteria for treatment discontinuation have already been described in previous work from our group [6].

Patients were included if they received at least one cycle of standard first-line doublet chemotherapy between March 2010 and January 2017 and provided written informed consent to treatment administration. Patients were excluded if their medical records lacked complete axial CT images of the abdominal and pelvic cavities.

### 2.2. Treatment

Standard first-line systemic chemotherapy schemes of the patients included in the study were either modified FOLFOX-6 regimen (mFOLFOX6) (two-weekly cycle of 5-FU 400 mg/m^2^ bolus on day 1 and 2400 mg/m^2^ continuous infusion on days 1–3 plus oxaliplatin 85 mg/m^2^ on day 1) or CapOX regimen (three-weekly cycle of oral capecitabine 1000 mg/m^2^ twice a day on days 1–14 plus oxaliplatin 130 mg/m^2^ on day 1), chosen individually for each patient on a clinical basis. Trastuzumab was added as per approved label in case of hyperexpression or amplification of Human Epidermal Growth Factor Receptor 2 (HER2). Discontinuation criteria were disease progression, unacceptable toxicity, or patient’s request to dismiss the treatment. Assessment of toxicity was performed according to Common Terminology Criteria for Adverse Events (CTCAE) version 5.0 by recording the highest grade of each adverse event throughout all cycles [12].

### 2.3. Efficacy and Outcome

Response Evaluation Criteria in Solid Tumors version 1.1 (RECIST 1.1) were used to assess the treatment efficacy through radiological exams performed every 8 to 12 weeks [13]. Progression-free survival (PFS) and overall survival (OS) were defined as the time from first-line chemotherapy initiation to the date of radiological/clinical progression or death from any cause and to the date of death or last follow-up, respectively.

### 2.4. Body Composition Parameters

**Figure 1:**
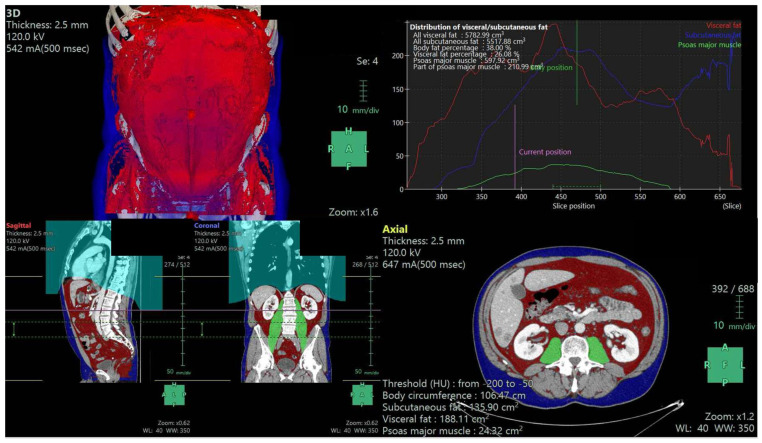
User interface in the extraction of 3D BCPs. The graphic shows the 3D (upper left panel) and 2D multiplanar reconstruction (lower panels) models of the semi-automated BCp extraction from a CT scan of the abdomen. Upper right panel shows the volume distribution of each major compartment (visceral fat, subcutaneous fat, and psoas muscle) throughout the z-axis. Note how variable the ratio between visceral and subcutaneous fat areas is, depending on what level is chosen to perform the measurement.

A trained radiologist (G.A.) with experience in oncologic imaging and blinded to clinical records evaluated baseline CT scans (thk = 2.5 to 3 mm) of our cohort, including the whole abdominal cavity. He extracted 2D and 3D body composition parameters (BCp) using semi-automated software (Synapse 3D v.4.4.001EU, Fujifilm, Singapore) after manually optimizing segmentation parameters. An example of the user interface for the BCp extraction is depicted in Figure 1. Extracted BCp included skeletal muscle index (SMI, defined as skeletal muscle area at L3 normalized by height squared), estimated total body fat-free mass (kg), total abdominal visceral and subcutaneous fat volumes (from diaphragm to symphysis pubis, cm^3^), psoas muscle volume (cm^3^), abdominal fat percentage (visceral + subcutaneous fat volumes/total abdominal cavity volume × 100), and visceral fat percentage (visceral fat volume/intraperitoneal + retroperitoneal cavity volumes × 100). According to the criteria proposed by Martin et al. [14], SMI was used to assess our patients as either sarcopenic or not. In detail, the cutoff used for defining sarcopenia in male patients was SMI < 43 cm^2^/m^2^ if BMI < 25 kg/m^2^ and SMI < 53 cm^2^/m^2^ if BMI ≥ 25 kg/m^2^ and in female patients as SMI < 41 cm^2^/m^2^ irrespective of BMI.

The ratio between visceral and subcutaneous fat volumes was calculated (3DVF/SF). Since no consistent threshold is available to date for assessing the prognosis of oncologic patients, we divided our cohort into quartiles, as previously performed in other studies of our [6] and other groups [15]. Patients were then classified according to 3D VF/SF used as a categorical variable, i.e., as having a “high ratio” if 3D VF/SF was greater than or equal to the median or a “low ratio” if less than the median.

Moreover, absolute measurements of subcutaneous fat, visceral fat, and psoas muscle volume were normalized on height cubed for all the patients, obtaining a “volume index” for each of these measurements. We did this based on the results obtained by [10] in investigating the prognosis of pancreatic cancer. Similar to the classification made in this study, we calculated absolute volume index distributions separately by sex. Then, we created “high” and “low” subgroups for each variable by comparing each individual with the median value of her/his sex.

### 2.5. Statistical Analysis

Continuous variables were compared across cohort subgroups using Mann–Whitney U test. Fisher’s exact test and Pearson’s chi-squared test were used for categorical variables with two and more than two categories, respectively.

The impact of 3D BCp on aGC patients’ survival was estimated through OS and PFS Kaplan–Meier tests and quantified using the log-rank test. Differences in the response to chemotherapy were assessed through Pearson’s chi-square test. A multivariate Cox proportional hazard model was used to identify independent prognostic factors in our cohort. All the analyses have been performed using Pandas (NumFOCUS, Austin, TX, USA) tool for Python 2.7.16.

## 3. Results

### 3.1. Comprehensive Characterization of aGC Patient Cohort: Clinical, Epidemiological, and Body Composition Profiles with Emphasis on Visceral and Subcutaneous Fat Ratios

Clinical and epidemiological data about our cohort are summarized in Table 1. The median age was 67 years (IQR: 59.25–74.75). Male sex was prevalent (45/65, 69%), and performance status (PS), measured according to the Eastern Cooperative Oncology Group (ECOG), was optimal (0) in 52% of patients. Neutrophil-to-lymphocyte and platelet-to-lymphocyte ratios, prognostic parameters for advanced gastric cancer [5,16], were over their specific cutoff values in 45 and 43% of patients, respectively. The primary tumor site was the esophagogastric junction and proximal and distal stomach in 38%, 35%, and 26% of cases, respectively.

Primary surgery with curative intention had been performed in 26% of patients. At first-line chemotherapy initiation, two-thirds of our patients had documented metastases in only one site among the liver, lymph nodes, lung, peritoneum, and bones. Lymph nodes were the most prevalent metastasis site (65% of patients), followed by the peritoneum (51%) and liver (38%). According to Martin’s criteria [14], 23 patients out of 58 (39.6%) were sarcopenic.

Median visceral and subcutaneous fat volumes were 2154 (IQR: 1450–3920) and 3296 (IQR: 1929–5367), respectively. The median visceral/subcutaneous 3D fat ratio was 0.837, which served as a cutoff for dividing our cohort into “Low” (*N* = 32) and “High” (*N* = 33) 3D fat ratio groups. The two groups were significantly different in sex distribution (fewer female subjects in the “high” group, *p* < 0.001) and primary tumor site (more proximal in the “high” group, *p* = 0.017). Moreover, the “high” group shows a significantly lower subcutaneous fat index (*p* < 0.001) and fat percentage in the abdomen (*p* = 0.011) and hints of a higher psoas muscle mass index (*p* = 0.054). No significant difference was found in the visceral fat index and visceral fat percentage between the two groups.

### 3.2. Prognostic Significance and Body Composition Parameters in aGC: Insights from Univariate and Multivariate Cox Regression Analyses

Out of 65 patients, 59 (91%) deceased, and 63 (97%) progressed after the first-line chemotherapy. Univariate Cox regression outputs are summarized in Table 2. OS was significantly associated with ECOG PS (*p* = 0.04), liver (*p* = 0.02) and bone (*p* = <0.005) metastasis, neutrophil-to-lymphocyte ratio (NLR) (*p* = 0.02), and 3D fat ratio (“high” vs. “low”, *p* = 0.02). PFS was predicted by ECOG PS (*p* = 0.02), NLR (*p* < 0.005), primary curative surgery (*p* = 0.07), and 3D fat ratio (*p* < 0.005). No influence on either PFS or OS was observed for any of the other 3D BCPs.

**Figure 2:**
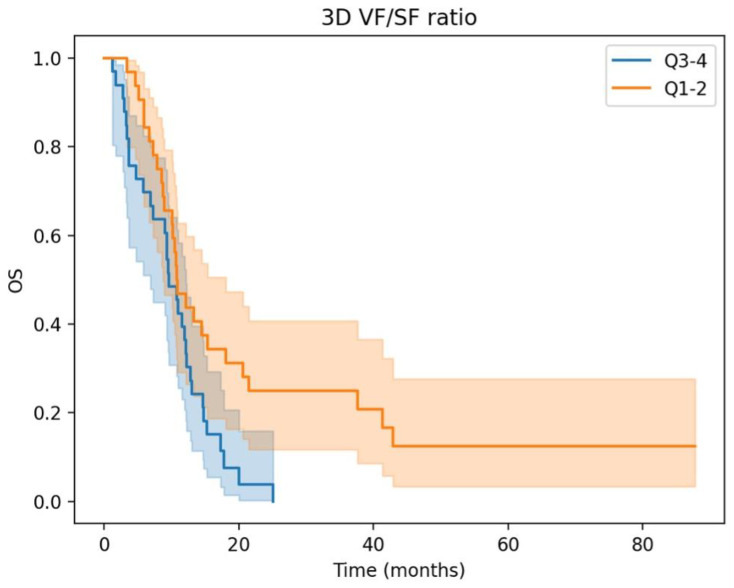
Overall survival is significantly lower in the “high” 3D fat ratio subgroup (log-rank *p* = 0.01).

**Figure 3:**
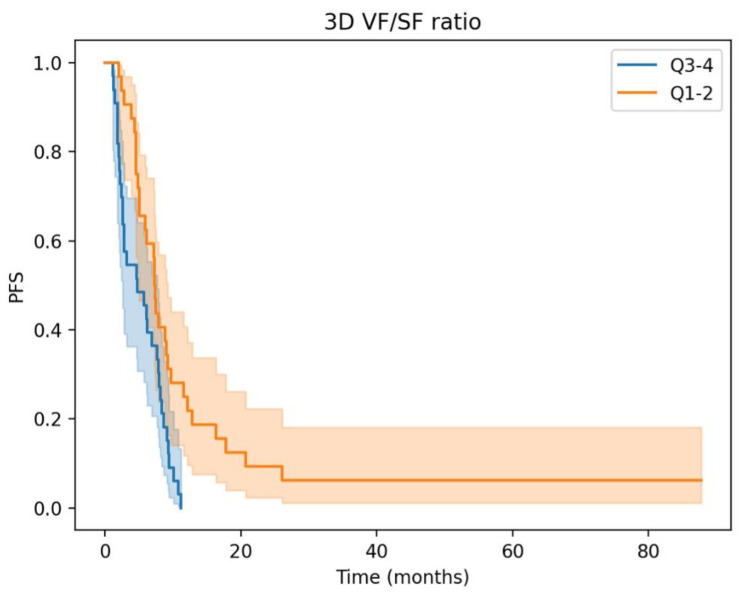
Progression-free survival is significantly lower in the “high” 3D fat ratio subgroup (log-rank *p* < 0.005).

Kaplan–Meier OS and PFS curves of the two 3D fat ratio subgroups are depicted in Figure 2 and Figure 3, respectively. The “High” 3D fat ratio group, corresponding to quartiles 3 and 4, shows a significant reduction in OS and PFS.

Multivariate Cox Regression analysis (Table 3) shows that PFS is predicted by ECOG PS (*p* = 0.05) and the 3D fat ratio (*p* = 0.01); OS is predicted by ECOG PS (*p* = 0.02), liver (*p* = 0.02) and bone (*p* < 0.005) metastasis, and the 3D fat ratio (*p* = 0.01). NLR could not be included in the model due to missing values in *N* = 8 patients.

### 3.3. Association of 3D Fat Ratio with Treatment Response in aGC: Insights from First-Line Chemotherapy Outcomes

**Figure 4:**
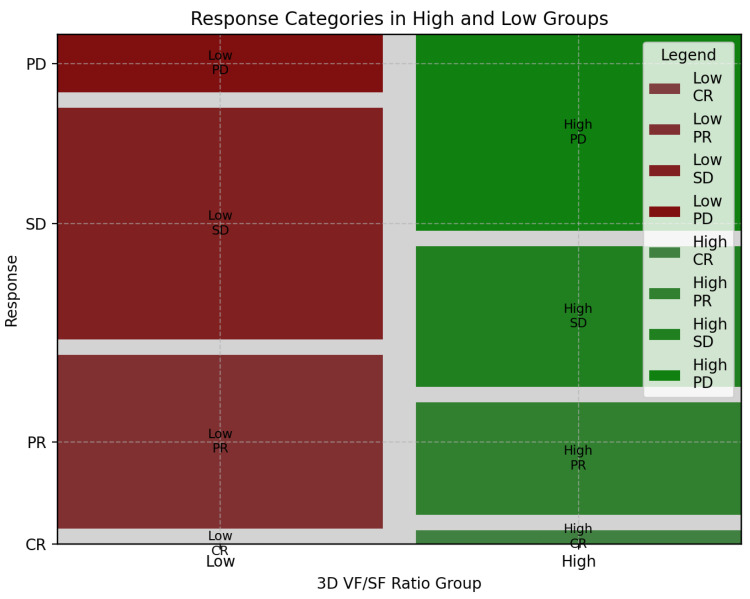
Best response to 1st-line chemotherapy in different 3D fat ratio subgroups.

**Figure 5:**
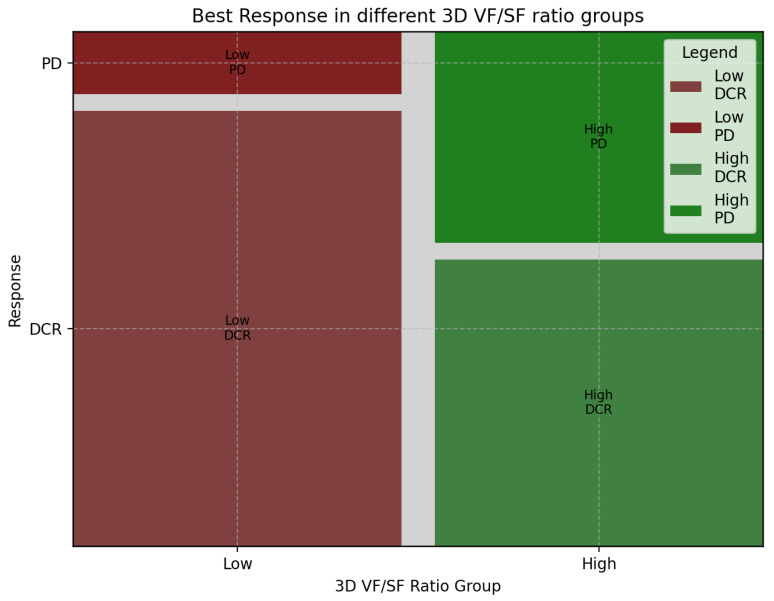
Disease control ratio in the 3D fat ratio subgroups.

The best response to first-line chemotherapy is summarized in Table 4. Progressive disease (PD) was observed in 18 cases (28%), divided into 14 high-ratio (42%) and 4 low-ratio (12%) patients. Stable disease (SD) was observed in 26 patients (40%). Among them, 10 belong to the high-ratio group (30%), and 16 to the low-ratio group (50%). A total of 20 patients (31%) showed partial response (PR): 8 from the high-ratio (24%) and 12 (37%) from the low-ratio group. Only one patient (high-ratio group) showed complete response (CR) after first-line chemotherapy. The distribution of the two groups across the best response categories is depicted in Figure 4. The chi-squared test was significant between the two distributions (*p* = 0.033). Accordingly, the disease control rate (DCR) was different between the two subgroups (Figure 5; chi-squared, *p* = 0.016).

### 3.4. Chemotherapy Tolerance and Adverse Events in aGC Patients: Impact of 3D Fat Ratio on Treatment Exposure and Side Effect Profile

Our patients received 2 to 13 cycles of chemotherapy (median = 8). Patient exposure to chemotherapy (expressed as the number of treatment cycles) was not significantly different between the low- and high-ratio groups (mean = 9 vs. 7.39, respectively, *p* = 0.051). Out of 65 patients, 13 (20%) underwent a dose reduction of chemotherapy, and 23 (35%) had to delay drug administration due to toxicity. In five cases (7.7%), chemotherapy was discontinued due to intolerable adverse effects. Dose reduction, administration delay, and discontinuation were not different between 3D fat ratio subgroups (*p* = 0.999, *p* = 0.999, and *p* = 0.200, respectively).

Incidence of nausea, vomiting, diarrhea, and hematologic adverse events was similar between the two subgroups. Neurotoxicity and mucositis were more prevalent in the low-ratio group (87.5% vs. 57.6%, *p* = 0.012, and 62.5% vs. 30.3%, *p* = 0.013, respectively). Overall severity of diarrhea and mucositis was also higher in the low-ratio group (*p* = 0.006).

## 4. Discussion

In our study, we show that the ratio of visceral and subcutaneous fat volumes is a strong and independent predictor of PFS and OS in patients with aGC treated with first-line palliative chemotherapy. In particular, survival outcomes were significantly worse in patients with a high visceral/subcutaneous fat volume ratio. Moreover, this subgroup showed a poor response to palliative chemotherapy in terms of lower DCR and higher PD proportions. This is in line with previous findings by our group regarding the surface ratio of the two fat compartments at L3 [6]. Yet, this study only found significance for a lower OS (*p* = 0.02) and PFS (*p* = 0.03) of the upper quartile of the VFA/SFA distribution. Our findings relate to the upper half of the VF/SF distribution, thus yielding prognostic value for a larger number of patients. In addition, the impact of 3D BCPs on aGC prognosis is statistically more significant (in terms of OS, *p* = 0.01, and PFS, *p* = 0.01) than 2D measurements.

Our findings overcome the intrinsic limitations of the previous 2D measurements given by spatial subsampling and account for interindividual variations in fat distribution along the longitudinal axis (see Figure 1). Moreover, direct and derived 3D BCPs may allow for a more straightforward implementation of automated parameter extraction, which could yield prognostic factors to clinical practice in real-time without requiring additional post-processing time.

Consistently with our finding that depletion of single fat compartments (visceral and subcutaneous) did not predict a worse outcome, we may argue that the mechanism through which the alteration of fat distribution affects aGC prognosis is not directly related to sarcopenia- or cachexia-induced weight loss but encompasses a more complex pathway.

In general, malignant tumors have been proven to alter glucose metabolism and induce peripheral insulin resistance (IR) [9], most likely due to long-term exposure to a pro-inflammatory molecular environment. Moreover, gastric cancer is specifically related to IR and C-peptide secretion [17]. In turn, IR and glucose metabolism disruption have been associated with inflammation through the action of several molecular mediators, including cytokines, Toll-like Receptors (TLR), and the IκB kinase-β (IKKβ)/NF-κB axis [18]. Visceral and subcutaneous fat measurements have been proven to be independently and positively correlated to IR [19]. However, there is evidence that visceral fat, different from subcutaneous fat, may promote carcinogenesis through the secretion of pro-inflammatory cytokines and free fatty acids, particularly in the obese [20,21]. Pro-inflammatory cytokines have also been found to be correlated with tumor growth, immune escape, and metastasis [22].

Less is known about the relative contribution of subcutaneous fat and about the interplay between the two compartments. A retrospective study conducted by Kaess et al. [23] on the Framingham Heart Study cohort showed that the visceral-to-subcutaneous fat volume ratio is a predictor of cardiometabolic risk, independent from visceral fat alone. The visceral-to-subcutaneous fat surface ratio at L4–L5 was found to be predictive of lower OS and PFS in rectal cancer patients treated with neoadjuvant chemoradiation and subsequent surgery [24]. In another study by Lee et al. [25], the subcutaneous fat area at L3 emerged as an independent predictor of survival in castration-resistant prostate cancer patients. Interestingly, in a large cohort of non-metastatic colorectal cancer patients treated with curative surgery, higher preoperative SFA was found to be predictive of better disease-free survival [26]. Together, these findings suggest that visceral/subcutaneous fat imbalance may represent an anthropometric reflection of underlying metabolic alterations with an impact on oncologic and non-oncologic patients. To shed light on this relation, future research will need to: (a) reach deeper knowledge of the subcutaneous fat metabolism; (b) assess the association of visceral/subcutaneous fat ratio with blood markers of visceral adiposity syndrome and inflammation; (c) investigate the differential impact of the two compartments on the response to chemotherapy; and (d) using large cohorts, delineate the context where their ratio is predictive of a worse outcome.

We did not observe any significant association between 2- or 3D parameters of muscle mass depletion and survival outcomes. This is consistent with previous findings from our group [6]. In the literature, the prognostic meaning of radiologically defined sarcopenia is well established in oncologic patients with different gastrointestinal and hepatobiliary malignancies [27,28,29,30,31] but becomes controversial when it comes specifically to aGC [32]. Kuwada et al. (2019) have reviewed the existing studies on this topic [8]. They conclude that radiological parameters of sarcopenia are globally associated with postoperative complications and poor survival, but with some differences among cohorts. Namely, the association with survival is evident in studies with smaller proportions of metastatic patients (stage ≥ 3 in 22% of the cohort [33]) or studies that excluded them at all [34,35,36] but could not be demonstrated in a cohort with 57.8% of patients above stage III [32]. This may be explained by the fact that in local and regional diseases, sarcopenia results from a reduction of dietary intake due to the mechanical effects of the malignancy on the gastrointestinal tract. In metastatic disease, this mechanism overlaps with cachexia, which is induced by systemic disease and pro-inflammatory state [37] and provokes a further loss of muscle mass. In our cohort, the proportion of metastatic patients was 77%, thus making it hard to distinguish the effects of the two overlapping pathophysiological mechanisms. Two studies conducted on metastatic GC patients failed to find any predictive value of SMI on survival [38,39] but found OS to be related to muscle mass loss through follow-up time and to skeletal muscle density, respectively. Another recent study found that SMI was predictive of OS in metastatic GC patients [25] but used Korean-specific cutoffs with considerable differences compared to the normative values in the Caucasian population. Globally, these findings seem to suggest that baseline measurement of skeletal muscle area is inadequate to describe the prognosis of aGC patients. However, to better elucidate the context in which sarcopenia can be a valid prognostic factor and to test the hypothesis that it may only be robust in regional disease, a large cohort should be enrolled with both non-metastatic and metastatic patients, and separate survival analysis should be run on each subgroup.

This study is not devoid of limitations. Firstly, the sample size is too small to facilitate subgroup analyses, thereby limiting our ability to investigate the pathophysiological basis of the observed results. The reliability and generalizability of our results will need to be confirmed by future studies with larger sample sizes. Secondly, the retrospective and observational design may be susceptible to selection bias among the enrolled patients. These constraints underscore the imperative for a large, prospective trial aimed at assessing the prognostic significance of body composition parameters in patients with aGC.

## 5. Conclusions

Patients with aGC and a higher visceral/subcutaneous fat volume ratio have shorter PFS and OS and show a worse response to first-line palliative chemotherapy. No prognostic value was found for muscle mass measurements. Further studies are required to clarify the biological and metabolic interplay between the two abdominal fat compartments.

## Figures and Tables

**Table 1:** Cohort characteristics, according to distribution of abdominal fat. Continuous data were compared using Mann–Whitney U test; categorical data were compared using Fisher’s exact test (2 categories) or Pearson’s chi-squared test (more than 2 categories).

Characteristics	All Patients (*N* = 65)	3D Fat Ratio Low (*N* = 32)	High (*N* = 33)	*p*
Age, years Median (range)	67 (59.25–74.75)	67 (61–74)	67 (57–75)	0.474
Sex (female/male)	19 (29)/46 (71)	17 (53)/15 (47)	2 (6)/31 (94)	<0.001
ECOG PS 0 vs. 1–2	30 (52)/28 (48)	16 (50)/16 (50)	14 (42)/19 (57)	0.622
Primary tumor site EGJ/GP/GD	25 (38)/23 (35)/17 (26)	7 (22)/13 (41)/12 (37)	18 (54)/10 (30)/5(15)	0.017
Primary tumor surgery (yes/no)	17 (26)	10 (31)	7 (21)	0.407
N° metastatic sites 0/1/ > =2/na	6 (9)/42 (65)/10 (15)/7 (11)	4 (12)/18 (56)/6 (19)/4 (12)	2 (6)/24(73)/4 (12)/3 (9)	0.561
Metastatic sites Liver Lung Peritoneum Lymph nodes Bones	25 (38) 6 (9) 33 (51) 42 (65) 6 (9)	13 (41) 2 (6) 17 (53) 20 (62) 3 (9)	12 (36) 4 (12) 16 (48) 22 (67) 3 (9)	0.801 0.673 0.805 0.798 0.999
HER2 mutant Yes/no/na	13 (20)/36 (55)/16 (25)	5 (16)/24 (75)/3 (50)	8 (24)/17 (51)/8 (24)	0.339
NLR > 3 Yes/no/na	29 (45)/28 (43)/8 (12)	15 (47)/13 (41)/4 (12)	14 (42)/15 (45)/4 (12)	0.793
PLR > 200 Yes/no/na	28 (43)/28 (43)/9 (14)	16 (50)/12 (38)/4 (12)	12 (36)/16 (48)/5 (15)	0.423
BMI <20/20–24.9/25–29.9/>30	14 (22)/32 (49)/15 (23)/4 (6)	5 (16)/17 (53)/8 (25)/2 (6)	9 (27)/15 (45)/7 (21)/2 (6)	0.724
SMI, median (range; SD) Female Male	48 (41–54; 9) 40 (36–45; 8) 51 (46–56; 7)	44.40 (37.97–54.42; 9.59) 40 (34–45; 8.39) 55 (46–64.5; 10.60)	48.42 (44.42–53.96) 42 48.48 (45.01–55.40; 7.83)	0.202
Visceral fat volume, median (range; SD)	2154 (1450 –3920; 1969)	1989 (1328–2949; 1500)	2843 (1556–4445; 2994)	0.094
Subcutaneous fat volume, median (range; SD)	3296 (1929–5367; 2694)	4623 (2754–6048; 2775)	2185 (1001–3857; 2169)	<0.001
Visceral fat index, median (range; SD)	438 (325–665; 387)	421 (347–583; 332)	530 (312–756; 434)	0.214
Subcutaneous fat index, median (range; SD)	617 (365–957; 517)	849 (605–1212; 546)	429 (248–660; 387)	<0.001
Psoas mass index, median (range; SD)	59 (46–75; 20)	55 (43–72; 18)	63 (48–82; 22)	0.054
Abdominal fat percentage, median (range, SD)	28.39 (21.17–35.55; 11.99)	30.81 (26.08–37.23; 9.51)	24.99 (16.80–33.90; 13.28)	0.011
Visceral fat percentage, median (range; SD)	26.39 (18.76–36.35; 12.65)	26.08 (20.08–31.04; 9.09)	27.36 (17.60–41.07; 15.36)	0.229

**Table 2:** Univariate Cox Regression.

Variable	PFS—HR (95% CI)	*p*	OS—HR (95% CI)	*p*
Age ≥ vs. <67 years	1.22 (0.74–2.01)	0.44	0.94 (0.56–1.59)	0.28
Sex Female vs. Male	0.98 (0.57–1.69)	0.94	1.03 (0.58–1.83)	0.91
ECOG PS >0 vs. =0	1.86 (1.12–3.09)	0.02	1.72 (1.02–2.89)	0.04
Primary tumor surgery (curative intention)	0.58 (0.33–1.03)	0.07	0.74 (0.41–1.33)	0.31
N° metastatic sites >1 vs. = 1	1.22 (0.61–2.44)	0.57	1.43 (0.69–2.95)	0.34
Metastatic sites Liver Peritoneum Lymph nodes Lungs Bones	0.74 (0.44–1.24) 0.96 (0.58–1.58) 1.16 (0.69–1.95) 1.59 (0.68–3.72) 1.61 (0.69–3.77)	0.25 0.88 0.58 0.28 0.27	0.54 (0.31–0.92) 1.30 (0.78–2.18) 1.17 (0.68–2.01) 1.25 (0.50–3.13) 3.76 (1.56–9.07)	0.02 0.32 0.56 0.64 <0.005
NLR > 3 Yes vs. no	2.35 (1.31–4.24)	<0.005	1.97 (1.12–3.48)	0.02
PLR > 200 Yes vs. no	1.13 (0.65–1.94)	0.67	1.45 (0.83–2.54)	0.19
SMI (Martin) Yes vs. no	0.92 (0.54–1.58)	0.76	1.06 (0.61–1.85)	0.82
Psoas volume index High vs. Low	0.88 (0.54–1.46)	0.63	1.04 (0.62–1.73)	0.89
Visceral fat volume index High vs. Low	1.11 (0.68–1.83)	0.67	1.22 (0.73–2.06)	0.45
Subcutaneous fat volume index High vs. Low	0.68 (0.41–1.11)	0.12	0.98 (0.59–1.64)	0.95
Abdominal fat percentage High vs. Low	0.95 (0.58–1.56)	0.83	1.02 (0.61–1.71)	0.94
Visceral fat percentage High vs. Low	0.84 (0.51–1.38)	0.49	0.82 (0.49–1.37)	0.44
3D Visceral/Subcutaneous fat ratio High vs. Low	2.20 (1.28–3.78)	<0.005	1.96 (1.13–3.38)	0.02

**Table 3:** Multivariate Cox Regression.

Variable	PFS—HR (95% CI)	*p*	OS—HR (95% CI)	*p*
ECOG PS	1.53 (1.00–2.33)	0.05	1.69 (1.07–2.66)	0.02
Liver metastasis	0.70 (0.39–1.24	0.22	0.50 (0.27–0.91)	0.02
Bone metastasis	1.68 (0.67–4.17)	0.27	3.96 (1.52–10.29)	<0.005
Primary surgery	0.63 (0.33–1.17)	0.14	0.67 (0.35–1.28)	0.23
High 3D fat ratio	2.09 (1.19–3.67)	0.01	2.16 (1.23–3.80)	0.01

**Table 4:** Best response to first-line chemotherapy.

Response	High-Ratio Group	Low-Ratio Group	Sum
CR	1	0	1
PR	8	12	20
SD	10	16	26
PD	14	4	18
Sum	33	32	65

## Data Availability

The data presented in this study are available on request from the corresponding author in an anonymized format. The data are not publicly available due to the clinical setting of their collection.
